# Efficacy and Safety of [^225^Ac]Ac-PSMA-617 Augmented [^177^Lu]Lu-PSMA-617 Radioligand Therapy in Patients with Highly Advanced mCRPC with Poor Prognosis

**DOI:** 10.3390/pharmaceutics13050722

**Published:** 2021-05-14

**Authors:** Florian Rosar, Jonas Krause, Mark Bartholomä, Stephan Maus, Tobias Stemler, Ina Hierlmeier, Johannes Linxweiler, Samer Ezziddin, Fadi Khreish

**Affiliations:** 1 Department of Nuclear Medicine, Saarland University, 66421 Homburg, Germany; florian.rosar@uks.eu (F.R.); s8jokrau@stud.uni-saarland.de (J.K.); mark.bartholomae@uks.eu (M.B.); stephan.maus@uks.eu (S.M.); tobias.stemler@uks.eu (T.S.); inamaria.hierlmeier@uks.eu (I.H.); samer.ezziddin@uks.eu (S.E.); 2 Department of Urology, Saarland University, 66421 Homburg, Germany; johannes.linxweiler@uks.eu

**Keywords:** metastatic castration-resistant prostate cancer, ^225^Ac and ^177^Lu, PSMA radioligand therapy, biochemical response, molecular imaging response, efficacy, toxicity

## Abstract

The use of ^225^Ac in prostate-specific membrane antigen (PSMA)-targeted radioligand therapy (RLT), either as monotherapy or in combination with ^177^Lu, is a promising therapy approach in patients with metastatic castration-resistant prostate carcinoma (mCRPC). In this study, we report the efficacy and safety of [^225^Ac]Ac-PSMA-617 augmented [^177^Lu]Lu-PSMA-617 RLT in ^177^Lu-naive mCRPC patients (*n* = 15) with poor prognosis (presence of visceral metastases, high total tumor burden with diffuse bone metastases or a short PSA doubling time of <2 months). Biochemical (by PSA serum value) and molecular imaging response (by [^68^Ga]Ga-PSMA-11 PET/CT) was assessed after two cycles of [^177^Lu]Lu-PSMA-617 RLT, with at least one [^225^Ac]Ac-PSMA-617 augmentation. In addition, PSA-based progression-free survival (PSA-PFS), overall survival (OS) and toxicity (according to CTCAE) were analyzed. We observed a biochemical- and molecular imaging-based partial remission in 53.3% (8/15) and 66.7% (10/15) of patients, respectively. The median PSA-PFS and OS was 9.1 and 14.8 months, respectively. No serious acute adverse events were recorded. Two out of fifteen patients experienced grade 3 anemia. No other grade 3/4 toxicities were observed. RLT-related xerostomia (grade 1/2) was recorded in 2/15 patients. Our data showed a high clinical efficacy with a favorable side effects profile of [^225^Ac]Ac-PSMA-617 augmented [^177^Lu]Lu-PSMA-617 RLT in this highly challenging patient cohort.

## 1. Introduction

Prostate carcinoma is currently ranked as the second most frequent malignancy and the fifth leading cause of cancer-related death in men worldwide [1]. Patients with metastatic prostate carcinoma are initially treated with androgen deprivation therapy (ADT), but a considerable number of patients ultimately reach the stage of metastatic castration-resistant prostate carcinoma (mCRPC) [2,3]. Bone and lymph node metastases are the most dominant, but visceral, especially liver metastases, are also quite frequent [4,5].

In the stage of mCRPC, taxane-based chemotherapy (docetaxel and cabazitaxel) [6,7], treatment with novel androgen axis drugs (NAAD) (abiraterone or enzalutamide) [8,9] and bone-seeking ^223^Ra therapy (Xofigo^®^) [10] are currently the standard treatment options [11], which are approved by the European Medicines Agency (EMA) and the US Food and Drug Administration (FDA). Recently, the EMA and FDA approved PARP-inhibitors (e.g., Olaparib) [12] for the treatment of mCRPC patients with alterations in DNA repair genes (e.g., BRCA 1/2 mutations).

If these treatments are ineffective, radioligand therapy targeting the prostate-specific membrane antigen (PSMA) is a promising therapy approach. PSMA, also known as folate hydrolase 1 (FOLH1) or glutamate carboxypeptidase II (GCPII), is one of the proteins overexpressed on the surface of prostate carcinoma cells [13]. PSMA radioligand therapy (PSMA-RLT) using the beta emitter lutetium-177 ([^177^Lu]Lu-PSMA-617 or [^177^Lu]Lu-PSMA-I&T) revealed encouraging data in several studies in mCRPC patients [14,15,16,17,18,19]. Both PSMA-targeted radioligands are currently being tested in phase III trials (e.g., VISION Trial (NCT03511664) and SPLASH Trial (NCT04647526)). Lutetium-177 (^177^Lu, half-life: 6.7 d) emits beta particles with moderate energies (E_max_ = 0.5 MeV), resulting in a particle range of about 2 mm, and a linear energy transfer (LET) of about 0.2 keV/µm in tissue [20]. PSMA-RLT using alpha emitters as actinium-225 (^225^Ac, half-life: 9.9 d), whose particles (E = 5.8 MeV) possess a shorter tissue range (<0.1 mm) and a higher LET (>50 keV/µm) [21,22], may have an advantage in comparison to PSMA-RLT with beta emitters. Recently, clinical studies using ^225^Ac-labeled PSMA-ligands ([^225^Ac]Ac-PSMA-617 or [^225^Ac]Ac-PSMA-I&T) have reported remarkable therapeutic results [23,24,25,26,27,28,29,30]. However, the stronger radiobiological effect of alpha particles also has implications to the organs at risk. Xerostomia seems to be the most prominent adverse effect of PSMA-RLT using ^225^Ac and may compromise the patients’ quality of life. Combining alpha emitters in adjusted doses as an augmentation to PSMA-RLT with beta emitters (so-called ‘tandem therapy’), may reduce these significant adverse effects in comparison to monotherapy using alpha emitters alone, while potentially increasing the therapeutic efficacy in comparison to monotherapy using beta emitters alone.

[^225^Ac]Ac-PSMA-617/[^177^Lu]Lu-PSMA-617 tandem therapy was reported as an effective treatment option in patients who exhibited progress or an insufficient response to [^177^Lu]Lu-PSMA-617 monotherapy [31,32]. Evaluation of this treatment approach combining alpha and beta RLT might thus be performed in other mCRPC patient cohorts. To the best of our knowledge, [^225^Ac]Ac-PSMA-617 as an augmentation to [^177^Lu]Lu-PSMA-617 RLT in ^177^Lu-naive mCRPC patients has not been reported to date. In this retrospective study, we report on the efficacy and safety profile of [^225^Ac]Ac-PSMA-617 augmentation in the initial phase of [^177^Lu]Lu-PSMA-617 RLT in patients with highly advanced mCRPC attributed to poor prognosis.

## 2. Materials and Methods

### 2.1. Patient Population

This retrospective study comprised *n* = 15 patients with highly advanced mCRPC who received [^225^Ac]Ac-PSMA-617 augmentation in the initial phase of [^177^Lu]Lu-PSMA-617 RLT. The initial phase was defined as the first two cycles of [^177^Lu]Lu-PSMA-617 RLT. Patients had to be in a highly advanced mCRPC setting with poor prognosis fulfilling at least one of the following criteria: (1) visceral metastases, (2) high total tumor burden with diffuse bone metastases or (3) short PSA doubling time (DT) of <2 months. Each patient received multiple therapies prior to PSMA-RLT, including ADT, NAAD, chemotherapy, Olaparib and ^223^Ra therapy. Detailed information on patient characteristics and pre-treatments is summarized in Table 1. All patients received at least two cycles of PSMA-RLT and were imaged by [^68^Ga]Ga-PSMA-11 PET/CT before and after two cycles of PSMA-RLT. PSMA-RLT was performed on a compassionate use basis under the German Pharmaceutical Act §13 (2b). All patients were treated within a prospective patient registry (REALITY Study, NCT04833517). Patients gave their consent after being fully informed about the risks and potential adverse effects of these procedures. Moreover, the patients agreed to the publication of the resulting data in accordance with the Declaration of Helsinki. The study was approved by the local Institutional Review Board (ethics committee permission number 140/17).

### 2.2. Treatment Details

All patients (*n* = 15) received two cycles of [^177^Lu]Lu-PSMA-617 with at least one [^225^Ac]Ac-PSMA-617 augmentation. The first (*n* = 7), the second (*n* = 3) or both (*n* = 5) of the two cycles of [^177^Lu]Lu-PSMA-617 were augmented with [^225^Ac]Ac-PSMA-617. [^225^Ac]Ac-PSMA-617 and [^177^Lu]Lu-PSMA-617 were synthesized analogously to published procedures [23,33] and administered during an inpatient stay according to German radiation protection regulations. The mean cumulative activity of [^177^Lu]Lu-PSMA-617 and [^225^Ac]Ac-PSMA-617 after the two initial PSMA-RLT cycles was 13.4 ± 2.6 GBq (corresponding to 169 ± 53 MBq/kg body weight (BW)) and 3.7 ± 1.7 MBq (corresponding to 45 ± 19 kBq/kg BW), respectively. The mean administered activity of [^177^Lu]Lu-PSMA-617 per cycle was 6.7 ± 1.8 GBq (corresponding to 84 ± 29 MBq/kg BW). The mean administered activity of [^225^Ac]Ac-PSMA-617 augmentation per cycle was 2.7 ± 1.1 MBq (corresponding to 33 ± 15 kBq/kg BW). Applied activities of both radioligands were individually chosen in consideration of each patient’s condition, the total tumor burden and the sites of metastases. Each patient received external cooling of the salivary glands and 1 L intravenous hydration (0.9% NaCl) 30 min before to two hours after radioligand administration.

### 2.3. Therapeutic Efficacy

Therapeutic efficacy was assessed through a change in biochemical and molecular imaging variables after the two cycles of PSMA-RLT. In addition, progression-free and overall survival were determined.

*Biochemical response rate.* PSA serum values were collected at the start of PSMA-RLT and a few weeks (mean 6 ± 2 weeks) after the second cycle of PSMA-RLT. Biochemical response was defined as a PSA reduction of 50% or more from baseline. Progression was defined by an increase of at least 25% and at least 2 ng/mL according to the PCWG3 guideline [34]. Stable disease was defined as a PSA change between −50% and 25%. 

*Molecular imaging response rate.* All patients were imaged by PSMA PET/CT mean 12 ± 14 days before the first and mean 6 ± 2 weeks after the second cycle of PSMA-RLT. [^68^Ga]Ga-PSMA-11 was used for imaging as it is currently the most widely used PET tracer in clinical routines and studies on prostate cancer [35]. PET/CT images were recorded on an EANM-accredited Biograph 40 mCT (Siemens Medical Solutions, Knoxville, TN, USA) with a mean administered activity of 124 ± 25 MBq [^68^Ga]Ga-PSMA-11 and an incubation time of approximately 60 min. PET data were acquired from vertex to mid-femur (3 min per bed position) and reconstructed using an iterative 3-dimensional ordered subset expectation maximization algorithm (3 iterations; 24 subsets; slice thickness 5 mm). Molecular imaging parameters as the whole-body total lesion PSMA (TLP) and molecular tumor volume (MTV) were determined by semi-automatic tumor segmentation using Syngo.Via (Enterprise VB 40B, Siemens, Erlangen, Germany). In accordance with Ferdinandus et al. [36], a threshold of standard uptake value (SUV) ≥3.0 was used for tumor segmentation. The physiological uptake of salivary glands, lacrimal glands, liver, spleen, intestine, kidney, ureter and bladder was manually excluded. Due to the intense uptake in the healthy liver, a threshold of 1.5 × SUV_mean_ of the normal liver tissue was applied for the segmentation of liver metastases. TLP was calculated as the summed products of volume and uptake (SUV_mean_) of all lesions, similar to the established parameter of total lesion glycolysis (TLG) in [^18^F]FDG PET/CT [37]. To avoid altering PSMA expression, ADT and NAAD were continued unchanged between both [^68^Ga]Ga-PSMA-11 PET/CT scans [38]. Modified PET response criteria in solid tumors (PERCIST) version 1.0 [39] were applied as follows: Molecular imaging-based partial remission represents a decrease of MTV or TLP > 30%. Progressive disease was defined as an increase in MTV or TLP > 30% or the appearance of any new lesion. A change in MTV or TLP in the range between +30% and −30% was considered as stable disease.

*Survival.* Analysis of progression-free survival (PFS) and overall survival (OS) based on the Kaplan–Meier method was performed using Prism 8 (GraphPad Software, San Diego, CA, USA). PFS was based on frequent measurements of the PSA serum value (PSA-PFS) and defined as the time interval from the start of PSMA-RLT to whichever came first: (1) evidence of PSA progression, (2) the last study visit or (3) death of any cause. OS was defined as the interval from the start of PSMA-RLT to the occurrence of any of the following: (1) death from any cause, (2) the last study visit or (3) initiation of a different treatment (e.g., chemotherapy). The cut-off follow-up date was 15th March 2021.

### 2.4. Safety

To assess hematotoxicity, blood tests, including hemoglobin, leukocytes and platelets, were performed before the start and after two cycles of PSMA-RLT (on the same days as the PSA serum values were measured). Renal toxicity was assessed by using the creatine-based estimated glomerular filtration rate (eGFR). Toxicity and adverse events were recorded and graded according to the Common Terminology Criteria for Adverse Events version 5.0 (CTCAE). Xerostomia was evaluated on patient reports via a questionnaire during hospitalization and at each outpatient visit. The questionnaire used was developed by our department and was based on CTCAE, including dry mouth feeling during the day, at night or while eating; swallowing problems; and intake alterations.

## 3. Results

### 3.1. Therapeutic Efficacy

#### 3.1.1. Biochemical Response Rate

**Figure 1:**
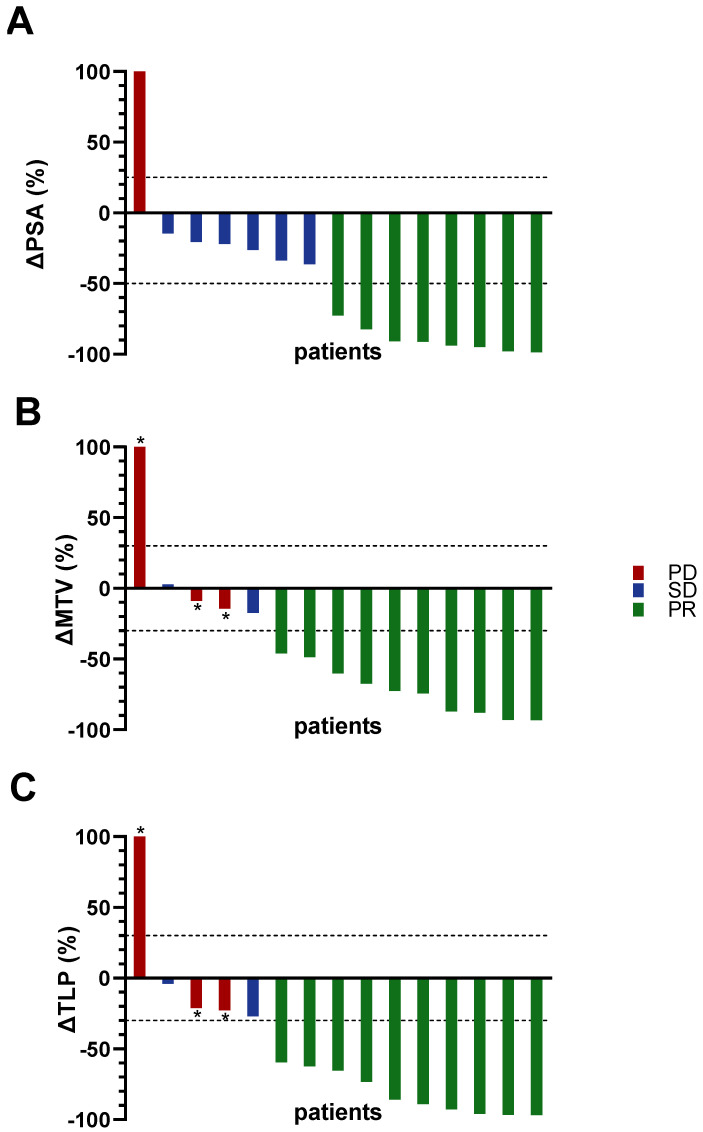
Waterfall plots of individual changes in (**A**): PSA serum value; (**B**): MTV; (**C**): TLP. Red: progressive disease (PD). Blue: stable disease (SD). Green: partial remission (PR). * Appearance of new metastases.

At the baseline of treatment, the mean PSA serum value was 667 ± 895 ng/mL (range: 58–3389 ng/mL). After two cycles of [^177^Lu]Lu-PSMA-617 RLT with at least one [^225^Ac]Ac-PSMA-617 augmentation, the mean PSA serum value was 249 ± 398 ng/mL (range: 1.4–1391 ng/mL). The median decrease was −72.6%. The detailed values of each patient are compiled in Table 2, and the relative changes are illustrated as a waterfall plot in Figure 1A.

Biochemical partial remission was observed in 8/15 (53.3%) patients, stable disease in 6/15 (40%) and progressive disease in 1/15 (6.7%).

#### 3.1.2. Molecular Imaging Response Rate

The total tumor burden was assessed by MTV and TLP in PET images. At baseline, the mean MTV and TLP were 1291 ± 1210 mL (range: 110–4002 mL) and 9558 ± 9476 mL × SUV (range: 636–34,273 mL × SUV), respectively. After two cycles of [^177^Lu]Lu-PSMA-617 RLT with at least one [^225^Ac]Ac-PSMA-617 augmentation, the mean MTV and TLP were 887 ± 1047 mL (range: 9–2747 mL) and 5214 ± 6381 mL × SUV (range: 36–16,688 mL × SUV), respectively. Individual values are summarized in Table 2. The median decreases in MTV and TLP were 60.1% and 65.4%, respectively. Figure 1B,C show the relative changes of each parameter for all patients. The results of response assessments using MTV or TLP were identical for all patients.

**Figure 2:**
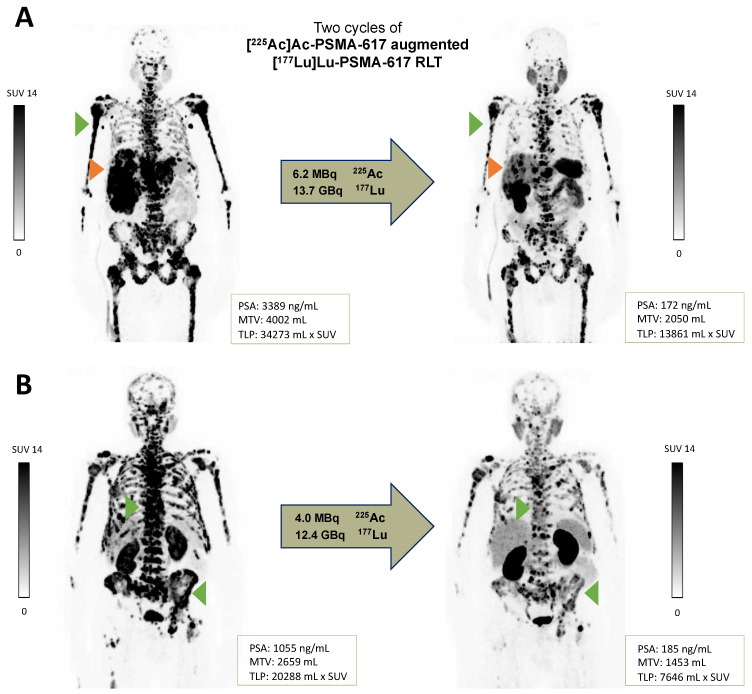
[^68^Ga]Ga-PSMA-11 PET/CT images of two mCRPC patients (**A**,**B**) at baseline and after two cycles of [^225^Ac]Ac-PSMA-617 augmented [^177^Lu]Lu-PSMA-617 RLT showing partial remission (exemplary: liver metastases, orange arrow; bone metastases, green arrow).

Molecular imaging partial remission was observed in 10/15 (66.7%) patients and stable disease in 2/15 (13.3%) patients. Progressive disease was recorded in 3/15 (20%) patients, all with the appearance of new metastases and in one with an additional increase in TLP/MTV > 30%. [^68^Ga]Ga-PSMA-11 PET/CT images of two responders with corresponding PSA, MTV and TLP values are shown in Figure 2. Molecular imaging and biochemical response assessment were concordant in 11/15 (73.3%) cases. The four discrepant cases were all assessed as stable diseases by PSA; however, two were categorized as partial remission and two as progressive disease (due to the appearance of new metastases) by molecular imaging.

#### 3.1.3. Survival

**Figure 3:**
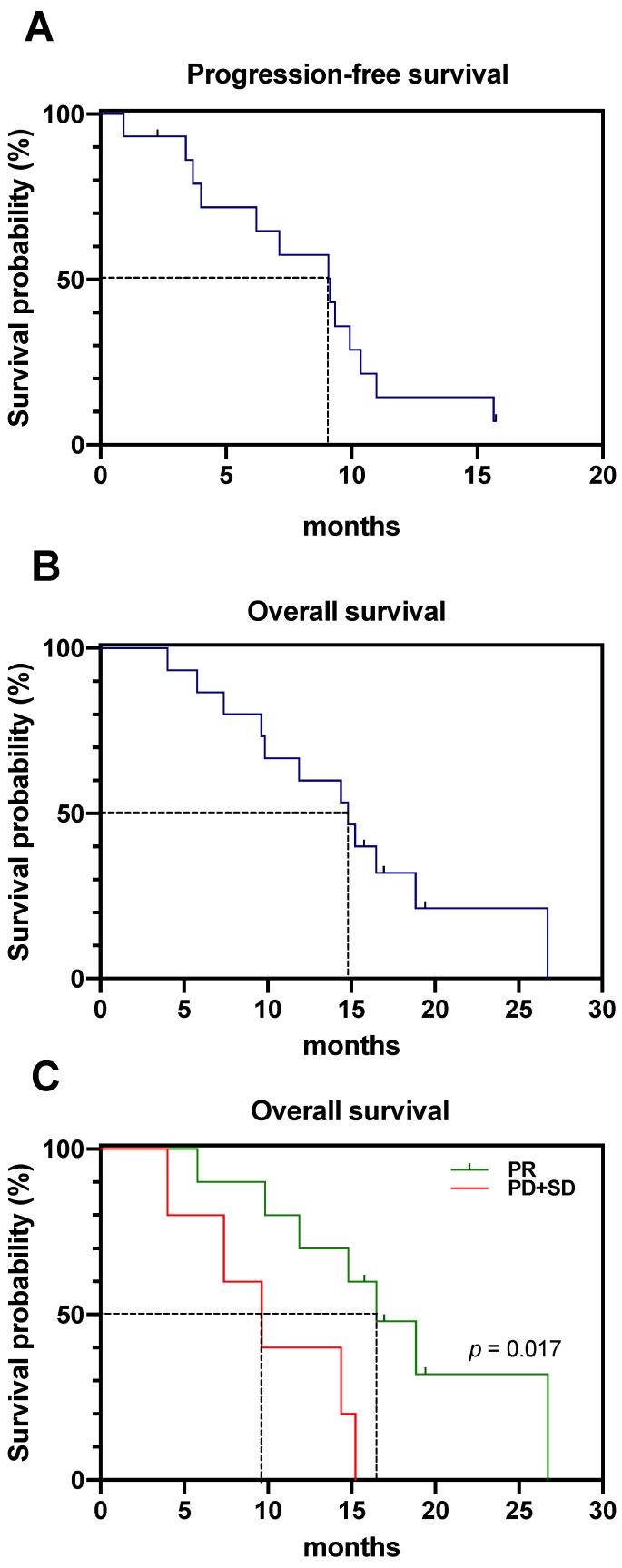
Kaplan–Meier curves of (**A**): PSA-based progression-free survival of the entire cohort; (**B**): overall survival of the entire cohort; (**C**): overall survival stratified by molecular imaging response (green: partial remission (PR); red: progressive disease (PD) or stable disease (SD)).

After the two cycles of PSMA-RLT, 13 patients continued PSMA-RLT with a median of two cycles (range: 1–6 cycles). Two additional [^225^Ac]Ac-PSMA-617 augmentations were given to 3/13 patients, and one additional [^225^Ac]Ac-PSMA-617 augmentation was given to 4/13 patients. From the date of initiating PSMA-RLT, the median follow-up time was 19.4 months. Fourteen out of fifteen patients (93.3%) exhibited disease progression during follow up. The median PSA-PFS was 9.1 months (CI: 3.7–10.4 months) (Figure 3A). At the end of the study, 12 patients had died due to mCRPC. The median OS was 14.8 months (CI: 9.6–16.9 months) (Figure 3B).

Patients showing partial remission by molecular imaging after two cycles reached a median OS of 16.5 months (CI: 9.8–19.4 months), whereas patients showing either stable or progressive disease only reached a median OS of 9.6 months (CI: 4.0–15.2 months) (Figure 3C). The difference in median OS was statistically significant (*p* = 0.017, log-rank test). In contrast, no significant difference (*p* = 0.116, log-rank test) in OS was noted between patients showing biochemical partial remission and those with biochemical stable or progressive disease. Median OS values were identical compared to molecular imaging, 9.6 months (CI: 4.0–26.7 months) for patients with biochemical stable or progressive disease and 16.5 months (CI: 9.8–19.4 months) for patients showing partial remission.

### 3.2. Safety Profile

**Figure 4:**
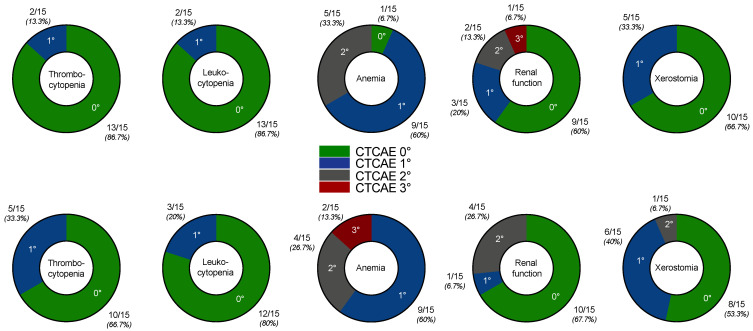
Graphical illustration of CTCAE grades for thrombocytopenia, leukocytopenia, anemia, renal function impairment and xerostomia at baseline (upper row) and after two cycles of [^177^Lu]Lu-PSMA-617 RLT with at least one [^225^Ac]Ac-PSMA-617 augmentation (lower row).

[^225^Ac]Ac-PSMA-617 augmented [^177^Lu]Lu-PSMA-617 RLT was well-tolerated, and no serious acute adverse events were recorded. All CTCAE grades for thrombocytopenia, leukopenia, anemia, renal function impairment and xerostomia before and after two cycles of [^177^Lu]Lu-PSMA-617 RLT with at least one [^225^Ac]Ac-PSMA-617 augmentation are compiled in Figure 4. Except in *n* = 2 patients, who experienced CTCAE 3° anemia, no other grade 3/4 toxicities were observed. Moderate adverse events (CTCAE 2°) attributed to the treatment were recorded in terms of anemia, renal function impairment and xerostomia in each *n* = 1 patient. Mild adverse events (CTCAE 1°) related to the treatment were observed for thrombocytopenia, lymphocytopenia, anemia and xerostomia in *n* = 3, *n* = 2, *n* = 1 and *n* = 1 patients, respectively. All other CTCAE grades remained unchanged or sporadically improved in comparison to baseline. Six out of fifteen patients (40%) did not experience any toxicity related to PSMA-RLT.

## 4. Discussion

The use of ^225^Ac in targeted PSMA-RLT, either as monotherapy or in combination with ^177^Lu-labeled PSMA-RLT as tandem approach, has achieved promising results in patients with mCRPC who have progressed on monotherapy with ^177^Lu [30,31,32]. In this study, we report the efficacy and safety of [^225^Ac]Ac-PSMA-617 augmented [^177^Lu]Lu-PSMA-617 RLT in ^177^Lu-naive mCRPC patients with poor prognosis, namely, the presence of visceral metastases, high total tumor burden with diffuse bone metastases or rapid PSA increase (DT < 2 months). Our data showed a high clinical efficacy with a favorable side effects profile of [^225^Ac]Ac-PSMA-617 augmented [^177^Lu]Lu-PSMA-617 RLT in this highly challenging patient cohort.

The presence of visceral metastases, especially liver metastases, is a strong adverse prognostic factor in patients with mCRPC [40,41,42]. Published data dealing with [^177^Lu]Lu-PSMA-617 RLT in mCRPC confirmed the negative prognostic impact of liver metastases [43,44,45]. Furthermore, high overall tumor burden, especially with diffuse bone metastases, is also considered to be a negative prognostic factor [46,47,48], and, in the majority of patients, leads to a rapid deterioration of the general patient condition. In addition, a short doubling time of PSA in principle implies a rapid progression and high aggressiveness of the tumor [48,49]. In an attempt to achieve a better outcome for patients with those negative prognostic factors, we intended to intensify the therapeutic effect of [^177^Lu]Lu-PSMA-617 RLT by [^225^Ac]Ac-PSMA-617 augmentation. The alpha particles emitted by ^225^Ac have a much higher LET than electrons emitted by ^177^Lu, leading to clusters of irreparable double-strand DNA breaks. In contrast, beta radiation by ^177^Lu alone produces primarily single-strand breaks, which are more easily repaired by cell mechanisms [22].

After two cycles of [^177^Lu]Lu-PSMA-617 RLT augmented with at least one cycle of [^225^Ac]Ac-PSMA-617, we observed a biochemical partial remission in 53.3% (8/15) of treated patients. Although our patient cohort represents an unfavorably selected mCRPC sample with poorer prognosis, the observed high biochemical response rate is comparable to response rates reported for various non-preselected mCRPC patient cohorts on two or three cycles of [^177^Lu]Lu-PSMA-617 monotherapy, although associated with less prognosis-worsening conditions (reported response rates: 47%–60%) [16,17,18,19]. Furthermore, molecular imaging-based partial remission determined by PSMA PET/CT was noted in 66.7% (10/15) of patients, again corresponding to published data from less challenging cohorts after either two or three cycles of [^177^Lu]Lu-PSMA-617 monotherapy [50,51]. These promising response rates in such highly challenging patients with markedly adverse prognostic factors appear to be the consequence of the additional radiobiological effect of the alpha radiation. 

In addition to response-based outcome measures, survival-based outcomes may provide a stronger indication of the efficacy of a new therapeutic approach. The median PSA-PFS of 9.1 months and median OS of 14.8 months are encouraging in the mentioned context and compare favorably with that of [^177^Lu]Lu-PSMA-617 monotherapy in non-preselected cohorts [52,53,54]. For example, in a prospective trial with *n* = 50 mCRPC patients treated with [^177^Lu]Lu-PSMA-617, a median PSA-PFS of 6.9 months and OS of 13.3 months was reached [54]. Only a few studies investigated [^177^Lu]Lu-PSMA-617 RLT in selected subgroups with impaired prognosis. Gafita et al. reported an OS of 11.6 months in a multicenter study of *n* = 43 mCRPC patients with diffuse bone marrow involvement [55]. Our group recently observed an OS of 11.7 months in a monocentric study of *n* = 28 mCRPC patients with liver metastases [56]. Due to different pre-selection criteria, resulting in inconsistent patient cohorts, a direct comparison seems inappropriate. However, the suggested improved survival achieved in the present study might be attributed to the benefit of combining alpha radiation with beta radiation for PSMA-RLT.

Another notable finding of our study was that early molecular imaging response to [^225^Ac]Ac-PSMA-617 augmented [^177^Lu]Lu-PSMA-617 RLT was significantly associated with OS. Patients showing progressive disease or stable disease on imaging after two cycles had shorter OS than those with partial remission. This is consistent with the results of our previous study [31] and highlights the potential role of PSMA PET/CT, in particular, the determination of total tumor burden, for therapy monitoring. The early identification of patients with worsening disease course and resistance to PSMA-targeted irradiation is essential, as the therapeutic strategy of these patients needs to be adjusted. However, it remains unknown whether the assessment of total tumor burden can be integrated in clinical routine due to its extensive time requirements.

Due to the short tissue range of alpha particles and the resulting low ‘crossfire’ effect, the addition of ^225^Ac did not substantially increase hematotoxicity. After two cycles, only 2/8 patients with diffuse bone metastases experienced grade 3 anemia. Both patients had existing grade 2 anemia at baseline. No other grade 3/4 hematotoxicities were noted. Additionally, no grade 3/4 xerostomia or renal function impairment was observed. Notably, only one patient experienced grade 2 and one grade 1 xerostomia, which was related to RLT. In particular, this low rate of xerostomia is most likely attributable to the lower administered activity of ^225^Ac compared to other studies in which [^225^Ac]Ac-PSMA-617 is applied as monotherapy. For ^225^Ac-augmentation, we applied a mean activity of 33 kBq/kg BW, which is one third of the recommended activity for the application of [^225^Ac]Ac-PSMA-617 as monotherapy [57]. A prospective study on [^225^Ac]Ac-PSMA-617 monotherapy by Yadav et al. reported grade 1/2 xerostomia in 29% of patients [25], and other retrospective studies observed considerably higher rates [26,27,28,29]. However, it should be noted that in these studies, some patients received more than two cycles.

As with all retrospective data, the promising results of this study should be confirmed by further studies, ideally in prospective randomized trials with larger patient cohorts. Although the current study focused on a challenging population of highly advanced mCRPC patients with poor prognosis, the efficacy and safety of this treatment approach in a non-preselected group of mCRPC patients is also worth assessing. Furthermore, a comparison study to [^177^Lu]Lu-PSMA-617 monotherapy in a prospective setting is needed to show whether the combination of ^225^Ac and ^177^Lu positively affects the outcome of mCRPC, as indicated in our study. In addition, the investigation of the response depending on the lesion sites would also be of high interest. For these purposes, we recommend future clinical studies on the combined use of ^225^Ac and ^177^Lu in PSMA-RLT in larger patient cohorts.

## 5. Limitations

When interpreting the reported results of this study, some limitations must be taken into account. The most important are the retrospective study design and the small number of patients. Further limitations concern the inhomogeneity resulting from a non-fixed activity, augmentation and treatment protocol.

## 6. Conclusions

[^225^Ac]Ac-PSMA-617 augmented [^177^Lu]Lu-PSMA-617 RLT is an effective treatment approach with a favorable toxicity profile in mCRPC patients with poor prognosis. These promising results should be confirmed by future, ideally prospective, studies consisting of large patient cohorts.

## Figures and Tables

**Table 1:** Patient characteristics.

Patient Characteristics	Value
**Age**	
Median (min.–max.) years	77 (57–88)
Age ≥ 70 years, % (*n*)	73 (11)
**PSA**, median (min.–max.) in (ng/mL)	272 (58–3389)
**Alkaline phosphatase**, median (min.–max.) in (U/L)	115 (8–1659)
**Hemoglobin**, median (min.–max.) in (g/dL)	11 (8–13)
**ECOG performance score category, % (*n*)**	
≤1	80 (12)
2	13 (2)
3	7 (1)
**Sites of metastases, % (*n*)**	
Bone	100 (15)
Lymph node	73 (11)
Liver	40 (6)
Lung	13 (2)
Other	7 (1)
**Prior therapies, % (*n*)**	
Prostatectomy	47 (7)
Radiation	53 (8)
ADT	100 (15)
Abiraterone or Enzalutamide	100 (15)
Abiraterone	80 (12)
Enzalutamide	87 (13)
Abiraterone and Enzalutamide	67 (10)
Chemotherapy	67 (10)
Docetaxel	67 (10)
Cabazitaxel	27 (4)
Docetaxel and Cabazitaxel	27 (4)
^223^Ra	20 (3)
Olaparib	13 (2)
**Adverse prognostic factors at baseline, % (*n*)**	
Visceral metastases	47 (7)
High total tumor burden with diffuse bone metastases	53 (8)
PSA DT < 2 months	67 (10)

**Table 2:** Individual values of PSA, MTV and TLP at baseline and after two cycles of [^177^Lu]Lu-PSMA-617 RLT with at least one [^225^Ac]Ac-PSMA-617 augmentation.

Patient Number	Baseline			After Two Cycles		
	PSA (ng/mL)	MTV (mL)	TLP (mL × SUV)	PSA (ng/mL)	MTV (mL)	TLP (mL × SUV)
1	58	110	636	37	101	491
2	822	2357	17,374	606	2420	16,688
3	1055	2695	20,288	185	1453	7646
4	66	137	1131	1	9	36
5	97	299	1518	77	39	215
6	3389	4002	34,273	172	2050	13,861
7	130	156	1295	12	11	53
8	317	479	2431	87	156	647
9	416	329	3732	26	131	1291
10	1630	2142	11,234	1391	1835	8841
11	272	306	3096	24	78	341
12	204	1128	6727	857	2747	15,497
13	210	2415	15,417	139	1993	11,260
14	127	1883	13,036	99	226	953
15	1214	930	11,179	17	65	387

## Data Availability

The datasets used and analyzed during the current study are available from the corresponding author on reasonable request.

## References

[1] Bray F., Ferlay J., Soerjomataram I., Siegel R.L., Torre L.A., Jemal A. (2018). Global Cancer Statistics 2018: GLOBOCAN Estimates of Incidence and Mortality Worldwide for 36 Cancers in 185 Countries. CA Cancer J. Clin..

[2] Kirby M., Hirst C., Crawford E.D. (2011). Characterising the Castration-Resistant Prostate Cancer Population: A Systematic Review: The Epidemiology of CRPC. Int. J. Clin. Pract..

[3] Watson P.A., Arora V.K., Sawyers C.L. (2015). Emerging Mechanisms of Resistance to Androgen Receptor Inhibitors in Prostate Cancer. Nat. Rev. Cancer.

[4] Pezaro C., Omlin A., Lorente D., Rodrigues D.N., Ferraldeschi R., Bianchini D., Mukherji D., Riisnaes R., Altavilla A., Crespo M. (2014). Visceral Disease in Castration-Resistant Prostate Cancer. Eur. Urol..

[5] Pond G.R., Sonpavde G., de Wit R., Eisenberger M.A., Tannock I.F., Armstrong A.J. (2014). The Prognostic Importance of Metastatic Site in Men with Metastatic Castration-Resistant Prostate Cancer. Eur. Urol..

[6] de Bono J.S., Oudard S., Ozguroglu M., Hansen S., Machiels J.-P., Kocak I., Gravis G., Bodrogi I., Mackenzie M.J., Shen L. (2010). Prednisone plus Cabazitaxel or Mitoxantrone for Metastatic Castration-Resistant Prostate Cancer Progressing after Docetaxel Treatment: A Randomised Open-Label Trial. Lancet.

[7] Tannock I.F., Horti J., Oudard S., James N.D., Rosenthal M.A. (2004). Docetaxel plus Prednisone or Mitoxantrone plus Prednisone for Advanced Prostate Cancer. N. Engl. J. Med..

[8] de Bono J.S., Logothetis C.J., Molina A., Fizazi K., North S., Chu L., Chi K.N., Jones R.J., Goodman O.B., Saad F. (2011). Abiraterone and Increased Survival in Metastatic Prostate Cancer. N. Engl. J. Med..

[9] Scher H.I., Fizazi K., Saad F., Taplin M.-E., Sternberg C.N., Miller K., de Wit R., Mulders P., Chi K.N., Shore N.D. (2012). Increased Survival with Enzalutamide in Prostate Cancer after Chemotherapy. N. Engl. J. Med..

[10] Parker C., Nilsson S., Heinrich D., Helle S.I., O’Sullivan J.M., Fosså S.D., Chodacki A., Wiechno P., Logue J., Seke M. (2013). Alpha Emitter Radium-223 and Survival in Metastatic Prostate Cancer. N. Engl. J. Med..

[11] Mottet N., van den Bergh R.C.N., Briers E., Van den Broeck T., Cumberbatch M.G., De Santis M., Fanti S., Fossati N., Gandaglia G., Gillessen S. (2021). EAU-EANM-ESTRO-ESUR-SIOG Guidelines on Prostate Cancer—2020 Update. Part 1: Screening, Diagnosis, and Local Treatment with Curative Intent. Eur. Urol..

[12] de Bono J., Mateo J., Fizazi K., Saad F., Shore N., Sandhu S., Chi K.N., Sartor O., Agarwal N., Olmos D. (2020). Olaparib for Metastatic Castration-Resistant Prostate Cancer. N. Engl. J. Med..

[13] Ghosh A., Heston W.D.W. (2004). Tumor Target Prostate Specific Membrane Antigen (PSMA) and Its Regulation in Prostate Cancer. J. Cell. Biochem..

[14] Hofman M.S., Violet J., Hicks R.J., Ferdinandus J., Thang S.P., Akhurst T., Iravani A., Kong G., Ravi Kumar A., Murphy D.G. (2018). [^177^Lu]-PSMA-617 Radionuclide Treatment in Patients with Metastatic Castration-Resistant Prostate Cancer (LuPSMA Trial): A Single-Centre, Single-Arm, Phase 2 Study. Lancet Oncol..

[15] Barber T.W., Singh A., Kulkarni H.R., Niepsch K., Billah B., Baum R.P. (2019). Clinical Outcomes of ^177^Lu-PSMA Radioligand Therapy in Earlier and Later Phases of Metastatic Castration-Resistant Prostate Cancer Grouped by Previous Taxane Chemotherapy. J. Nucl. Med..

[16] Ahmadzadehfar H., Eppard E., Kürpig S., Fimmers R., Yordanova A., Schlenkhoff C.D., Gärtner F., Rogenhofer S., Essler M. (2016). Therapeutic Response and Side Effects of Repeated Radioligand Therapy with ^177^Lu-PSMA-DKFZ-617 of Castrate-Resistant Metastatic Prostate Cancer. Oncotarget.

[17] Rahbar K., Ahmadzadehfar H., Kratochwil C., Haberkorn U., Schäfers M., Essler M., Baum R.P., Kulkarni H.R., Schmidt M., Drzezga A. (2017). German Multicenter Study Investigating ^177^Lu-PSMA-617 Radioligand Therapy in Advanced Prostate Cancer Patients. J. Nucl. Med..

[18] Rasul S., Hacker M., Kretschmer-Chott E., Leisser A., Grubmüller B., Kramer G., Shariat S., Wadsak W., Mitterhauser M., Hartenbach M. (2020). Clinical Outcome of Standardized ^177^Lu-PSMA-617 Therapy in Metastatic Prostate Cancer Patients Receiving 7400 MBq Every 4 Weeks. Eur. J. Nucl. Med. Mol. Imaging.

[19] Fendler W.P., Reinhardt S., Ilhan H., Delker A., Böning G., Gildehaus F.J., Stief C., Bartenstein P., Gratzke C., Lehner S. (2017). Preliminary Experience with Dosimetry, Response and Patient Reported Outcome after ^177^Lu-PSMA-617 Therapy for Metastatic Castration-Resistant Prostate Cancer. Oncotarget.

[20] Kassis A.I. (2008). Therapeutic Radionuclides: Biophysical and Radiobiologic Principles. Semin. Nucl. Med..

[21] Ferrier M.G., Radchenko V. (2019). An Appendix of Radionuclides Used in Targeted Alpha Therapy. J. Med. Imaging Radiat. Sci..

[22] Juzeniene A., Stenberg V.Y., Bruland Ø.S., Larsen R.H. (2021). Preclinical and Clinical Status of PSMA-Targeted Alpha Therapy for Metastatic Castration-Resistant Prostate Cancer. Cancers.

[23] Kratochwil C., Bruchertseifer F., Giesel F.L., Weis M., Verburg F.A., Mottaghy F., Kopka K., Apostolidis C., Haberkorn U., Morgenstern A. (2016). ^225^Ac-PSMA-617 for PSMA-Targeted α-Radiation Therapy of Metastatic Castration-Resistant Prostate Cancer. J. Nucl. Med..

[24] Sathekge M., Bruchertseifer F., Knoesen O., Reyneke F., Lawal I., Lengana T., Davis C., Mahapane J., Corbett C., Vorster M. (2019). ^225^Ac-PSMA-617 in Chemotherapy-Naive Patients with Advanced Prostate Cancer: A Pilot Study. Eur. J. Nucl. Med. Mol. Imaging.

[25] Yadav M.P., Ballal S., Sahoo R.K., Tripathi M., Seth A., Bal C. (2020). Efficacy and Safety of ^225^Ac-PSMA-617 Targeted Alpha Therapy in Metastatic Castration-Resistant Prostate Cancer Patients. Theranostics.

[26] Sathekge M., Bruchertseifer F., Vorster M., Lawal I.O., Knoesen O., Mahapane J., Davis C., Reyneke F., Maes A., Kratochwil C. (2020). Predictors of Overall and Disease-Free Survival in Metastatic Castration-Resistant Prostate Cancer Patients Receiving ^225^Ac-PSMA-617 Radioligand Therapy. J. Nucl. Med..

[27] Zacherl M.J., Gildehaus F.J., Mittlmeier L., Boening G., Gosewisch A., Wenter V., Schmidt-Hegemann N.-S., Belka C., Kretschmer A., Casuscelli J. (2020). First Clinical Results for PSMA Targeted Alpha Therapy Using ^225^Ac-PSMA-I&T in Advanced MCRPC Patients. J. Nucl. Med..

[28] van der Doelen M.J., Mehra N., van Oort I.M., Looijen-Salamon M.G., Janssen M.J.R., Custers J.A.E., Slootbeek P.H.J., Kroeze L.I., Bruchertseifer F., Morgenstern A. (2020). Clinical Outcomes and Molecular Profiling of Advanced Metastatic Castration-Resistant Prostate Cancer Patients Treated with ^225^Ac-PSMA-617 Targeted Alpha-Radiation Therapy. Urol. Oncol..

[29] Satapathy S., Mittal B.R., Sood A., Das C.K., Singh S.K., Mavuduru R.S., Bora G.S. (2020). Health-Related Quality-of-Life Outcomes with Actinium-225-Prostate-Specific Membrane Antigen-617 Therapy in Patients with Heavily Pretreated Metastatic Castration-Resistant Prostate Cancer. Indian J. Nucl. Med..

[30] Feuerecker B., Tauber R., Knorr K., Heck M., Beheshti A., Seidl C., Bruchertseifer F., Pickhard A., Gafita A., Kratochwil C. (2021). Activity and Adverse Events of Actinium-225-PSMA-617 in Advanced Metastatic Castration-Resistant Prostate Cancer After Failure of Lutetium-177-PSMA. Eur. Urol..

[31] Rosar F., Hau F., Bartholomä M., Maus S., Stemler T., Linxweiler J., Ezziddin S., Khreish F. (2021). Molecular Imaging and Biochemical Response Assessment after a Single Cycle of [^225^Ac]Ac-PSMA-617/[^177^Lu]Lu-PSMA-617 Tandem Therapy in MCRPC Patients Who Have Progressed on [^177^Lu]Lu-PSMA-617 Monotherapy. Theranostics.

[32] Khreish F., Ebert N., Ries M., Maus S., Rosar F., Bohnenberger H., Stemler T., Saar M., Bartholomä M., Ezziddin S. (2020). ^225^Ac-PSMA-617/^177^Lu-PSMA-617 Tandem Therapy of Metastatic Castration-Resistant Prostate Cancer: Pilot Experience. Eur. J. Nucl. Med. Mol. Imaging.

[33] Kratochwil C., Giesel F.L., Stefanova M., Benešová M., Bronzel M., Afshar-Oromieh A., Mier W., Eder M., Kopka K., Haberkorn U. (2016). PSMA-Targeted Radionuclide Therapy of Metastatic Castration-Resistant Prostate Cancer with ^177^Lu-Labeled PSMA-617. J. Nucl. Med..

[34] Scher H.I., Morris M.J., Stadler W.M., Higano C., Basch E., Fizazi K., Antonarakis E.S., Beer T.M., Carducci M.A., Chi K.N. (2016). Trial Design and Objectives for Castration-Resistant Prostate Cancer: Updated Recommendations From the Prostate Cancer Clinical Trials Working Group 3. J. Clin. Oncol. Off. J. Am. Soc. Clin. Oncol..

[35] Fendler W.P., Eiber M., Beheshti M., Bomanji J., Ceci F., Cho S., Giesel F., Haberkorn U., Hope T.A., Kopka K. (2017). ^68^Ga-PSMA PET/CT: Joint EANM and SNMMI Procedure Guideline for Prostate Cancer Imaging: Version 1.0. Eur. J. Nucl. Med. Mol. Imaging.

[36] Ferdinandus J., Violet J., Sandhu S., Hicks R.J., Ravi Kumar A.S., Iravani A., Kong G., Akhurst T., Thang S.P., Murphy D.G. (2020). Prognostic Biomarkers in Men with Metastatic Castration-Resistant Prostate Cancer Receiving [^177^Lu]-PSMA-617. Eur. J. Nucl. Med. Mol. Imaging.

[37] Boellaard R., Delgado-Bolton R., Oyen W.J.G., Giammarile F., Tatsch K., Eschner W., Verzijlbergen F.J., Barrington S.F., Pike L.C., Weber W.A. (2015). FDG PET/CT: EANM Procedure Guidelines for Tumour Imaging: Version 2.0. Eur. J. Nucl. Med. Mol. Imaging.

[38] Rosar F., Dewes S., Ries M., Schaefer A., Khreish F., Maus S., Bohnenberger H., Linxweiler J., Bartholomä M., Ohlmann C. (2020). New Insights in the Paradigm of Upregulation of Tumoral PSMA Expression by Androgen Receptor Blockade: Enzalutamide Induces PSMA Upregulation in Castration-Resistant Prostate Cancer Even in Patients Having Previously Progressed on Enzalutamide. Eur. J. Nucl. Med. Mol. Imaging.

[39] Wahl R.L., Jacene H., Kasamon Y., Lodge M.A. (2009). From RECIST to PERCIST: Evolving Considerations for PET Response Criteria in Solid Tumors. J. Nucl. Med..

[40] Whitney C.A., Howard L.E., Posadas E.M., Amling C.L., Aronson W.J., Cooperberg M.R., Kane C.J., Terris M.K., Freedland S.J. (2017). In Men with Castration-Resistant Prostate Cancer, Visceral Metastases Predict Shorter Overall Survival: What Predicts Visceral Metastases? Results from the SEARCH Database. Eur. Urol. Focus.

[41] Shou J., Zhang Q., Wang S., Zhang D. (2018). The Prognosis of Different Distant Metastases Pattern in Prostate Cancer: A Population Based Retrospective Study. Prostate.

[42] Gandaglia G., Karakiewicz P.I., Briganti A., Passoni N.M., Schiffmann J., Trudeau V., Graefen M., Montorsi F., Sun M. (2015). Impact of the Site of Metastases on Survival in Patients with Metastatic Prostate Cancer. Eur. Urol..

[43] Ahmadzadehfar H., Schlolaut S., Fimmers R., Yordanova A., Hirzebruch S., Schlenkhoff C., Gaertner F.C., Awang Z.H., Hauser S., Essler M. (2017). Predictors of Overall Survival in Metastatic Castration-Resistant Prostate Cancer Patients Receiving [^177^Lu]Lu-PSMA-617 Radioligand Therapy. Oncotarget.

[44] Satapathy S., Mittal B.R., Sood A. (2020). Visceral Metastases as Predictors of Response and Survival Outcomes in Patients of Castration-Resistant Prostate Cancer Treated With ^177^Lu-Labeled Prostate-Specific Membrane Antigen Radioligand Therapy: A Systematic Review and Meta-Analysis. Clin. Nucl. Med..

[45] Kessel K., Seifert R., Schäfers M., Weckesser M., Schlack K., Boegemann M., Rahbar K. (2019). Second Line Chemotherapy and Visceral Metastases Are Associated with Poor Survival in Patients with MCRPC Receiving ^177^Lu-PSMA-617. Theranostics.

[46] Seifert R., Kessel K., Schlack K., Weber M., Herrmann K., Spanke M., Fendler W.P., Hadaschik B., Kleesiek J., Schäfers M. (2020). PSMA PET Total Tumor Volume Predicts Outcome of Patients with Advanced Prostate Cancer Receiving [^177^Lu]Lu-PSMA-617 Radioligand Therapy in a Bicentric Analysis. Eur. J. Nucl. Med. Mol. Imaging.

[47] Perez-Lopez R., Lorente D., Blackledge M.D., Collins D.J., Mateo J., Bianchini D., Omlin A., Zivi A., Leach M.O., de Bono J.S. (2016). Volume of Bone Metastasis Assessed with Whole-Body Diffusion-Weighted Imaging Is Associated with Overall Survival in Metastatic Castration-Resistant Prostate Cancer. Radiology.

[48] Moreira D.M., Howard L.E., Sourbeer K.N., Amarasekara H.S., Chow L.C., Cockrell D.C., Pratson C.L., Hanyok B.T., Aronson W.J., Kane C.J. (2017). Predicting Time from Metastasis to Overall Survival in Castration-Resistant Prostate Cancer: Results From SEARCH. Clin. Genitourin. Cancer.

[49] Miyazawa Y., Sekine Y., Shimizu N., Takezawa Y., Nakamura T., Miyao T., Nakayama H., Kurihara S., Syuto T., Nomura M. (2019). An Exploratory Retrospective Multicenter Study of Prognostic Factors in MCRPC Patients Undergoing Enzalutamide Treatment: Focus on Early PSA Decline and Kinetics at Time of Progression. Prostate.

[50] Michalski K., Mix M., Meyer P.T., Ruf J. (2019). Determination of Whole-Body Tumour Burden on [^68^Ga] PSMA-11 PET/CT for Response Assessment of [^177^Lu] PSMA-617 Radioligand Therapy: A Retrospective Analysis of Serum PSA Level and Imaging Derived Parameters before and after Two Cycles of Therapy. Nuklearmedizin.

[51] Grubmüller B., Senn D., Kramer G., Baltzer P., D’Andrea D., Grubmüller K.H., Mitterhauser M., Eidherr H., Haug A.R., Wadsak W. (2019). Response Assessment Using ^68^Ga-PSMA Ligand PET in Patients Undergoing ^177^Lu-PSMA Radioligand Therapy for Metastatic Castration-Resistant Prostate Cancer. Eur. J. Nucl. Med. Mol. Imaging.

[52] Ahmadzadehfar H., Rahbar K., Baum R.P., Seifert R., Kessel K., Bögemann M., Kulkarni H.R., Zhang J., Gerke C., Fimmers R. (2021). Prior Therapies as Prognostic Factors of Overall Survival in Metastatic Castration-Resistant Prostate Cancer Patients Treated with [^177^Lu] Lu-PSMA-617. A WARMTH Multicenter Study (the 617 Trial). Eur. J. Nucl. Med. Mol. Imaging.

[53] Meyrick D., Gallyamov M., Sabarimurugan S., Falzone N., Lenzo N. (2021). Real-World Data Analysis of Efficacy and Survival After Lutetium-177 Labelled PSMA Ligand Therapy in Metastatic Castration-Resistant Prostate Cancer. Target. Oncol..

[54] Violet J., Sandhu S., Iravani A., Ferdinandus J., Thang S.-P., Kong G., Kumar A.R., Akhurst T., Pattison D.A., Beaulieu A. (2020). Long-Term Follow-up and Outcomes of Retreatment in an Expanded 50-Patient Single-Center Phase II Prospective Trial of ^177^Lu-PSMA-617 Theranostics in Metastatic Castration-Resistant Prostate Cancer. J. Nucl. Med..

[55] Gafita A., Fendler W.P., Hui W., Sandhu S., Weber M., Esfandiari R., Calais J., Rauscher I., Rathke H., Tauber R. (2020). Efficacy and Safety of ^177^Lu-Labeled Prostate-Specific Membrane Antigen Radionuclide Treatment in Patients with Diffuse Bone Marrow Involvement: A Multicenter Retrospective Study. Eur. Urol..

[56] Khreish F., Kochems N., Rosar F., Sabet A., Ries M., Maus S., Saar M., Bartholomä M., Ezziddin S. (2021). Response and Outcome of Liver Metastases in Patients with Metastatic Castration-Resistant Prostate Cancer (MCRPC) Undergoing ^177^Lu-PSMA-617 Radioligand Therapy. Eur. J. Nucl. Med. Mol. Imaging.

[57] Kratochwil C., Bruchertseifer F., Rathke H., Bronzel M., Apostolidis C., Weichert W., Haberkorn U., Giesel F.L., Morgenstern A. (2017). Targeted α-Therapy of Metastatic Castration-Resistant Prostate Cancer with ^225^Ac-PSMA-617: Dosimetry Estimate and Empiric Dose Finding. J. Nucl. Med..

